# Transcript Level Responses of *Plasmodium falciparum* to Antimycin A

**DOI:** 10.1016/j.protis.2012.01.003

**Published:** 2012-09

**Authors:** Sarah J. Tarr, R. Ellen R. Nisbet, Christopher J. Howe

**Affiliations:** Department of Biochemistry, University of Cambridge, Tennis Court Road, Cambridge, Cambridgeshire, CB2 1QW, United Kingdom

**Keywords:** DHOD, dihydroorotate dehydrogenase, IMM, inner mitochondrial membrane, LD, lethal dose, mtETC, mitochondrial electron transport chain, qRT-PCR, quantitative reverse transcription polymerase chain reaction, *Plasmodium*, mitochondria, antimycin A, retrograde signalling.

## Abstract

The mitochondrial electron transport chain is essential to *Plasmodium* and is the target of the antimalarial drug atovaquone. The mitochondrial genomes of *Plasmodium* sp. are the most reduced known, and the majority of mitochondrial proteins are encoded in the nucleus and imported into the mitochondrion post-translationally. Many organisms have signalling pathways between the mitochondria and the nucleus to regulate the expression of nuclear-encoded mitochondrially-targeted proteins, for example in response to mitochondrial dysfunction. We have studied the transcript profiles of synchronous *Plasmodium falciparum* treated with an LD_50_ concentration of the complex III inhibitor antimycin A, to investigate whether such pathways exist in the parasite. There was a broad perturbation of gene expression. The differentially expressed genes were enriched for transcripts encoding proteins involved in invasion, stress response, nucleotide biosynthesis and respiration. Some effects were attributable to a delay in the gene expression phase of drug-treated parasites. However, our data indicated regulation of mitochondrial stress response genes and genes involved in pyrimidine biosynthesis, implying the existence of a signalling pathway from the mitochondrion to the nucleus.

## Introduction

*Plasmodium* sp. mitochondria are the site of a number of biochemical reactions including an unusual, bifurcated tricarboxylic acid (TCA) cycle (Olszewski et al. 2010), iron-sulphur cluster biogenesis, ubiquinone synthesis and stages of haem synthesis (van Dooren et al. 2006). Mitochondria are typically the site of ATP synthesis via the mitochondrial electron transport chain (mtETC); sequential redox reactions drive the translocation of protons across the inner mitochondrial membrane (IMM) generating membrane potential across the IMM which is harnessed for the synthesis of ATP via ATP synthase. However, the *Plasmodium* mtETC is not believed to contribute to ATP production during blood stage malaria infection (Fry et al. 1990). A functional mtETC is nonetheless essential and the Q_o_ site of the cytochrome *bc*_1_ complex is the target of the antimalarial drug atovaquone (Fry and Pudney 1992). Treatment of parasites with compounds such as atovaquone results in loss of membrane potential (Srivastava et al. 1997) and killing of the parasite. The mtETC of *Plasmodium* sp. has a number of unusual features including a single subunit, non-proton translocating NADH dehydrogenase and a cytochrome *c* oxidase subunit 2 that is split into two proteins (Gardner et al. 2002). The *Plasmodium* mtETC has five sources of electrons that can reduce ubiquinone; the aforementioned NADH dehydrogenase, succinate dehydrogenase, FAD-linked glycerol-3-phosphate dehydrogenase, malate:quinone oxidoreductase (which replaces malate dehydrogenase in the TCA cycle (van Dooren et al. 2006)) and dihydroorotate dehydrogenase (DHOD). DHOD is central to pyrimidine biosynthesis and, like most eukaryotic DHODs, donates its electrons to coenzyme Q. These electrons are then fed into the mtETC. Transfection of *P. falciparum* D10 parasites with a fumarate-dependent DHOD from yeast gave rise to atovaquone-resistant parasites (Painter et al. 2007), presumably due to the loss of dependence of pyrimidine biosynthesis on releasing electrons into the cytochrome *bc*_1_ complex via coenzyme Q. Furthermore, these parasites were also resistant to other *bc*_1_ complex inhibitors; the IC_50_ of the Q_i_ site inhibitor antimycin A rose from 129.4 nM to over 33000 nM, indicating the specificity of this drug for the *bc*_1_ complex in *P. falciparum*. Further evidence for this specificity comes from the observation that parasites selected for resistance against one *bc*_1_ complex inhibitor became resistant to a number of others (including antimycin A) while remaining sensitive to inhibitors of other metabolic pathways (Smilkstein et al. 2008).

The majority of *Plasmodium* mitochondrial proteins are encoded in the nucleus and imported into the mitochondrion post-translationally and the *Plasmodium* mitochondrial genome is the most reduced known (Barbrook et al. 2010). In many organisms, the expression of a number of these nuclear genes is subject to transcriptional regulation; in mammalian systems, subunits of all mtETC components (including ATP synthase) are subject to such control, for example throughout the cell cycle, or in response to temperature change or mitochondrial dysfunction (Scarpulla 2002). Furthermore, genes for import proteins and chaperones such as Tom20 and mtCpn10 are also subject to transcriptional regulation (Scarpulla 2002). mtETC components can be regulated by other means such as phosphorylation and allosteric mechanisms. Regulation of cytochrome *c* oxidase (complex IV) and cytochrome *c* has received attention. Complex IV is regulated by membrane potential, phosphorylation and allosterically by ATP, and cytochrome *c* is inhibited by phosphorylation (Hüttemann et al. 2007).

Cellular status and mitochondrial dysfunction signal changes in nuclear gene expression allowing the maintenance of essential metabolic pathways (Epstein et al. 2001; Hüttemann et al. 2007; Scarpulla 2002). Signalling from mitochondria to the nucleus has been well studied in yeast. One key pathway depends on a heterodimeric transcription factor consisting of Rtg1p and Rtg3p, along with its positive regulator, Rtg2p. As well as regulating basal expression, during times of mitochondrial perturbation (for example, in cells lacking mitochondrial DNA) these proteins are required for the enhanced expression of genes involved in maintaining glutamate production (Liu and Butow 2006). Yeast cells treated with the mtETC inhibitor antimycin A exhibit altered expression of genes linked to the maintenance of glutamate synthesis. Additionally, some elements of the antimycin A response in yeast are independent of these proteins; in particular, numerous transporters and permeases exhibit upregulation independent of the Rtg proteins (Epstein et al. 2001).

It is not known whether regulation of mtETC components occurs at either the protein- or transcript-level in *Plasmodium*. Our own searches of the *P. falciparum* genome failed to identify orthologues of components of the typical RTG-dependent mitochondria-nucleus signalling pathway. However, given the essential nature of the *Plasmodium* mtETC, we postulated that despite the organism's ‘hard wired’ patterns of gene expression (Ganesan et al. 2008), transcriptional regulation may occur to maintain mtETC function in response to mitochondrial dysfunction. As antimycin A was sufficient to induce a retrograde signalling response in yeast (Epstein et al. 2001), we studied global gene expression responses of *P. falciparum* to antimycin A. While sensitivity of *P. falciparum* to antimycin A is well documented, there is much discrepancy over the lethal concentrations of the drug in different strains. Consequently, we initially established the LD_50_ concentration of antimycin A across one full lifecycle in *P. falciparum* 3D7 parasites. Subsequently, we assessed global changes in gene expression in response to the drug using microarrays. A large number of genes exhibited differential expression upon drug treatment, including genes involved in multiple metabolic pathways. In absolute terms, we detected little induction of stress response genes. However, there appeared to be an enrichment of transcripts for stress response genes relative to their representation within the parasite genome. We also noted changes in expression of genes encoding cytoskeletal and invasion related proteins, which could be attributed to a delay in the gene expression profiles of the parasites. An enrichment of differentially expressed transcripts for proteins involved in respiration and the upregulation of transcripts encoding mitochondrial chaperones was in keeping with the effects of mtETC inhibition, such as production of reactive oxygen species. Given the presumed inhibitory effects of antimycin A on dihydroorotate dehydrogenase (due to prevention of regeneration of oxidised ubiquinone by the *bc*_1_ complex) it was also interesting to observe enrichment among upregulated genes of a number of transcripts for enzymes involved in nucleotide biosynthesis and in particular, pyrimidine biosynthesis. These data support the vital nature of this pathway to parasite survival. Furthermore, these data indicate that communication from the mitochondrion to the nucleus can occur in *P. falciparum* in response to mitochondrial dysfunction. While the mechanisms underlying this pathway are unclear, evidence for interorganellar communication in *Plasmodium* raises the prospects of novel drug targets for future investigation.

## Results

### Dose-dependent Effects of Antimycin A Treatment on the Growth of *P. falciparum* in Vitro

**Figure 1:**
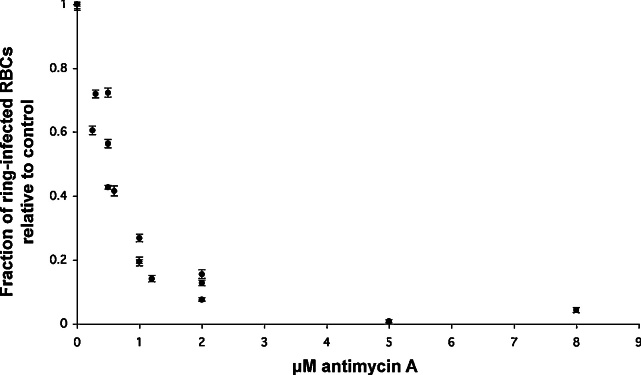
Infection of RBCs with ring stage parasites relative to controls after 48 hours incubation in the presence of varying concentrations of antimycin A. Error bars represent the combined standard deviation of proportions of parasitaemia in treated and control samples.

Published LD_50_ values of antimycin A in *P. falciparum* range from 13 nM in the D6 strain (Smilkstein et al. 2008) to 5.5 μM in the FCC strain (Geary et al. 1989). We sought to ascertain the LD_50_ concentration of antimycin A in 3D7 parasites. We were interested in the drug concentration sufficient to prevent progression of 50% of 3D7 parasites through one full cell cycle, rather than assessing inhibition of metabolism (such as DNA synthesis, which could be directly affected by the influence of the drug on pyrimidine biosynthesis). To determine LD_50_ under the conditions used here, synchronous, ring-stage parasites were treated for 48 hours with a range of concentrations of antimycin A. Parasite survival was defined as the proportion of parasites to complete one lifecycle successfully relative to untreated controls (Fig. 1). Only ring stage parasites were counted. We have used this simple technique to determine lethality over one lifecycle for other drugs (Tarr et al. 2011) and found our results to be consistent with those of other laboratories using both radiolabelled hypoxanthine uptake and DNA-binding dye assays (data not shown). The dose response curve in Figure 1 suggested an LD_50_ concentration of 600 nM antimycin A. This LD_50_ concentration is unlikely to lead to off-target effects, since parasites with resistance to a range of complex III inhibitors had an increased LD_50_ for antimycin A in excess of 2500 nM (Smilkstein et al. 2008). At 600 nM antimycin A, there was little observable phenotypic effect on growth over the first 24 hours of the parasite lifecycle (not shown). Consequently, a 24 hour, 600 nM antimycin A treatment was chosen for global gene expression analysis.

### Global Responses of *P. falciparum* to Antimycin A

**Figure 2:**
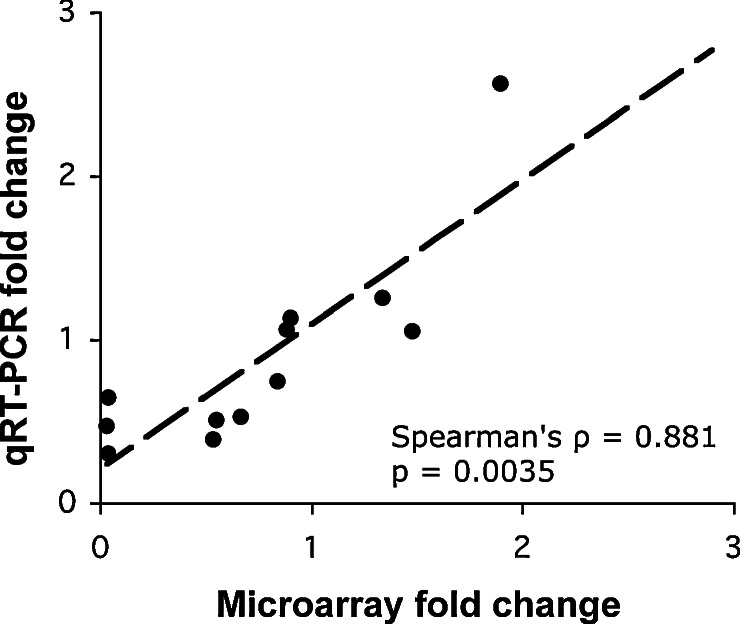
Correlation of microarray and qRT-PCR fold change values.

We used microarrays to characterise the global gene expression response of *P. falciparum* to 600 nM antimycin A over 24 hours. The data obtained were processed using GCRMA and analysed for differential gene expression using LIMMA. Based on a cut-off threshold of Bonferroni-Holm adjusted p < 0.001, 475 genes were classified as being differentially expressed between antimycin A-treated and untreated cultures. qRT-PCR analysis of twelve transcripts showed a strong correlation with the microarray data (Fig. 2; Spearman's ρ = 0.881, p = 0.0035).

**Figure 3:**
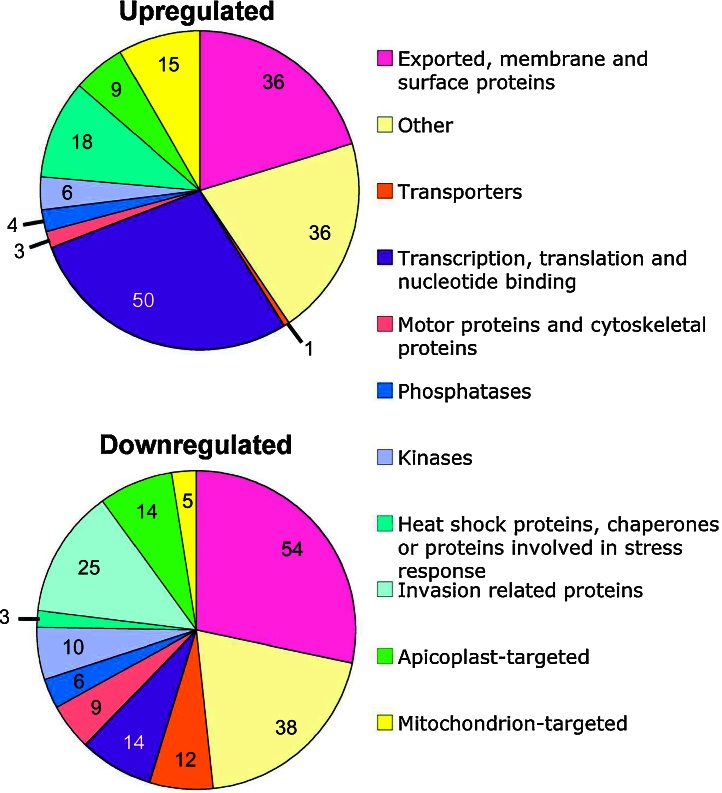
Functional categorisation of up- and downregulated genes differentially expressed upon treatment of *P. falciparum* with antimycin A (excluding proteins of unknown function), p < 0.001. Numbers reflect total numbers of differentially expressed genes within each category.

Two hundred and fifteen genes were found to be upregulated and 260 genes were found to be downregulated. The fold changes in expression were small; the median increase in expression was 2.485 fold and the median decrease in expression was 0.200 fold. 107 genes encoded proteins of unknown function. The remaining genes were placed into functional categories. Upregulated genes are shown in Supplementary Table 2 and downregulated genes are shown in Supplementary Table 3. The categorisations of differentially expressed up- and downregulated genes (excluding those of unknown function) are shown in Figure 3.

### Gene Ontology Enrichment Analysis

We sought to assess whether particular functional groups of genes were enriched within the dataset of differentially expressed genes relative to their representation within the parasite genome. We used the online gene ontology enrichment analysis tool of DAVID (Database for Annotation, Visualization and Integrated Discovery; (Huang da et al. 2009a, b), which supports analysis of *Plasmodium* gene IDs, to detect enrichment of particular biological terms among the differentially expressed genes. The upregulated and downregulated gene lists were loaded into the DAVID interface and subjected to functional annotation clustering in order to evaluate the representation of biological terms, using default parameters. The clustered groups are listed in Table 1; enrichment scores greater than 1 (bold in Table 1) correspond to p-value < 0.1.

The functional clustering indicated enrichment among upregulated genes of transcripts encoding chaperones and stress response proteins, as well as transcripts encoding proteins involved in nucleotide biosynthesis/transcription. Among downregulated genes, there was enrichment of transcripts encoding cytoskeletal, as well as invasion-related proteins. Furthermore, there was an enrichment of genes encoding proteins involved in respiration.

### Genes Encoding Mitochondrially-targeted Proteins

Only a small proportion of the differentially expressed genes encoded proteins targeted to the mitochondrion. Of the nineteen differentially expressed genes within this category, 14 were upregulated and 5 were downregulated. Upregulated genes encoded import proteins and chaperones involved in protein import, while downregulated genes encoded proteins involved in energy metabolism, indicating a functional distinction between up- and downregulated genes encoding mitochondrially-targeted proteins. While the absolute number of differentially expressed genes encoding mitochondrially-targeted proteins was small, the gene ontology enrichment analysis nonetheless indicated that there was enrichment among downregulated genes of transcripts encoding proteins involved in respiration. Among the five downregulated transcripts encoding mitochondrially-targeted proteins were genes encoding two subunits of cytochrome *c* oxidase (*coxIIb* by 0.48 fold and *coxVIb* by 0.47 fold), the putative ubiquinone-binding, 14 kDa subunit of complex III (PF10_0120, 0.56 fold) and citrate synthase (0.48 fold). However, no other TCA cycle components were found among the 475 differentially expressed genes. The upregulated genes included mitochondrial import proteins TIM44 (5.4 fold), GrpE (2.9 fold), Hsp60 (2.7 fold) and TIM23 (3.5 fold). Genes encoding prohibitin and Bcs1 were also upregulated. These are believed to act as chaperones for respiratory proteins. Furthermore, a 10 kDa chaperonin (*Pf*Cpn10) was upregulated (by 5.4 fold), as were mtHsp90 (3.3 fold) encoded by PF11_0188 and mtHsp70 (3.1 fold). A single cysteine desulphurase (IscS) was upregulated by 4.5 fold; other components of the iron-sulphur cluster biogenesis pathway were not differentially expressed. Three genes encoding mitochondrial ribosomal proteins were upregulated as well as PFL1185c, which encodes the enzyme responsible for insertion of haem into cytochrome *c*.

Genes for apicoplast-targeted proteins were not over-represented among the differentially expressed genes, in spite of the close association between mitochondrion and apicoplast (Howe and Purton 2007). Twenty-three differentially expressed genes (∼ 5%) encoded proteins predicted to be apicoplast-targeted. Of these, nine were upregulated and 14 were downregulated. Five upregulated genes encoded proteins involved in the expression and maturation of plastid proteins; two were proteins involved in tRNA synthesis, as well as a fMet deformylase (1.4 fold increase), an aminopeptidase (2.0 fold increase) and the apicoplast cochaperonin (*Pf*Cpn20; 1.9 fold increase). *Pf*Cpn20 plays a role in the maturation of plastid proteins. The gene encoding *Pf*Cpn60 with which it functions was not found to be upregulated. PFB0270w, an iron-sulphur assembly protein described as SufE, was upregulated by 1.9 fold. However, genes for other components of the Suf pathway were not regulated. PF13_0157 (which encodes one of two ribose-phosphate pyrophosphokinases in *P. falciparum*) was also upregulated by 2.4 fold. These enzymes provide 5-phospho-D-ribose 1-pyrophosphate for purine and pyrimidine biosynthesis. Of the 13 downregulated genes encoding apicoplast-targeted proteins, 8 were of unknown function.

### Genes Encoding Stress Response Proteins and Chaperones

Our analysis of enriched biological terms within the differentially expressed genes indicated an enrichment of genes encoding stress response proteins and chaperones among the upregulated transcripts. Twenty-one heat shock, chaperone and stress response protein genes were differentially expressed. Eighteen of these were upregulated, of which 17 were upregulated by greater than 1.5 fold and ten by greater than 2 fold. These included genes for glutaredoxin, glutathione synthase, one thioredoxin-like protein and multiple proteins annotated as heat shock proteins (or associated proteins) or as having DnaJ domains. In addition two genes encoding proteins involved in ubiquitin metabolism showed increased expression. The three downregulated genes in this category encoded the hsp90 co-chaperone p23 (by 0.57 fold), a putative thioredoxin (0.11 fold) and a putative ubiquitin transferase (0.077 fold).

We observed a predominant downregulation of genes encoding transporters. Thirteen genes encoding transporters showed differential expression. However, only a single (nucleoside) transporter gene was upregulated while the remainder (of varying function) were downregulated; these included two drug/metabolite transporters, a Na^+^/H^+^ antiporter and Ca^2+^and two Cu^2+^ ion transporters. Gene ontology enrichment analysis did not identify terms within this category as being significantly enriched among the dataset. However, transporter proteins were among the classifications identified by DAVID as enriched, albeit with p-values > 0.1.

### Invasion-related Proteins

Twenty-five genes involved in invasion were downregulated, while there were no upregulated genes in this category. Indeed, the gene ontology enrichment analysis identified invasion-related terms as being significantly enriched among the downregulated transcripts. Seven genes for rhoptry proteins were found to be differentially expressed, as were a number of genes encoding components of the motor complex required for invasion. These included GAP50 (0.43 fold), GAP45 (0.25 fold), myosin A tail interacting protein (0.15 fold) and multiple membrane skeletal proteins (0.097 – 0.10 fold). Five reticulocyte-binding proteins and three erythrocyte-binding antigens were also downregulated, all to less than 0.2 fold.

In keeping with the downregulation of invasion-related genes, nine of the 12 genes encoding motor proteins or cytoskeletal proteins were downregulated. Furthermore, functional terms linked to the cytoskeleton were identified as significantly enriched among downregulated transcripts. The downregulated transcripts within this category included genes involved in the formation of/interaction with actin filaments, as well as genes for myosin E and myosin D.

### Exported and Surface Proteins

Ninety genes encoding surface or exported proteins belonging to larger gene families exhibited differential expression and, for the majority of transcripts, changes were greater than ± 50%. Thirty-six were upregulated and 54 were downregulated. Eight merozoite surface protein genes were differentially expressed, all of which were downregulated. A number of single members of large multigene families (e.g. hyp) were also differentially expressed.

### Other Features of the Gene Expression Response to Antimycin A

Genes encoding phosphatases and kinases were considered as candidate genes with an involvement in intracellular signalling upon drug treatment. Ten genes encoding phosphatases were differentially expressed upon antimycin A treatment. Four showed increased expression between 1.8 and 3.3 fold (comprising two genes for protein phosphatases, an exopolyphosphatase and diadenosine tetraphosphatase). The 6 downregulated phosphatase genes encoded protein phosphatases and an inorganic pyrophosphatase.

Sixteen kinase genes showed altered expression upon antimycin A treatment. Of the six upregulated kinase genes, four encoded FIKK kinases (MAL7P1.144, PF10_0380, MAL7P1.175 and PFL0040c). Genes encoding protein kinase 6 (MAL13P1.185) and an adenylate kinase (PFD0755c) also exhibited increased expression. Downregulated kinase genes included three for serine/threonine protein kinases, three for calcium-dependent protein kinases, cGMP- and cAMP-dependent protein kinases as well as a gene for an uncharacterised protein kinase (encoded by PF13_0166).

Sixty-four of the genes differentially expressed upon antimycin A treatment were involved in transcription, translation or nucleotide binding. Of these, 50 were upregulated i.e. almost 25% of all upregulated genes. The genes within this category included transcripts for ten ribosomal proteins or proteins involved in ribosome biogenesis, six subunits of RNA polymerases I, II and III, two ApiAP2 proteins (PF10_0075 by 1.6 fold, and PFL1075w by 1.6 fold) and eight RNA helicases. Fourteen genes encoding proteins involved in transcription, translation or nucleotide binding were downregulated. Among these were four genes encoding proteins involved in the regulation of transcription; PfSir2A encoded by PF13_0152 was downregulated 0.20 fold, ADA2 transcriptional activator was downregulated 0.73 fold and two ApiAP2 proteins encoded by PFF0200c (0.16 fold) and PF11_0091 (0.49 fold). PFF0200c is known to be involved in telomeric maintenance (Flueck et al. 2010). Three genes encoding zinc finger proteins were also downregulated. Furthermore, functional terms linked to transcription and nucleotide biosynthesis were identified as significantly enriched among upregulated transcripts by the gene ontology enrichment analysis. Additional terms such as translation, tRNA and rRNA metabolism, were also identified by DAVID as showing some enrichment among upregulated genes, albeit with p-values higher than 0.1.

Seventy-four genes were defined as ‘other-metabolism’ as they did not fit into any of the alternative categories. Thirty-six genes were upregulated including two encoding proteins involved in pyridoxal 5-phosphate biosynthesis. Genes encoding proteins involved in pyrimidine metabolism were also upregulated, for example cytidine triphosphate synthetase (5.0 fold), cytidine/deoxycytidylate deaminase (4.0 fold), orotate phosphoribosyltransferase (3.4 fold) and aspartate carbamoyltransferase (2.4 fold). Ribose-phosphate pyrophosphokinase (which contributes to pyrimidine and purine biosynthesis but is predicted to be apicoplast targeted) was also upregulated by 2.2 fold.

Thirty-eight genes encoding proteins involved in additional metabolic pathways were downregulated. The proteins encoded by these genes were of varying function but included PfSUB1 and PfSUB2 subtilisin-like proteases and two serine repeat antigens. PfSUB1 is involved in the cleavage of SERAs prior to egress (Yeoh et al. 2007) and of MSP complexes prior to invasion (Koussis et al. 2009).

## Discussion

We have investigated the transcript-level effects of treatment with the mtETC inhibitor antimycin A on gene expression in *P. falciparum*. Twenty-four hours of treatment with an LD_50_ concentration of the drug led to the differential expression of a large number of genes and these were categorised into functional groups. Similar proportions of genes exhibited up- and downregulation. However, the distribution of genes among functional categories differed between the up- and downregulated genes.

Gene ontology enrichment analysis using DAVID indicated that there was enrichment among upregulated transcripts for genes involved in the stress response and nucleotide biosynthesis, while downregulated transcripts were enriched for genes involved in invasion and the cytoskeleton.

### General Responses to Antimycin A

In general, there was a broad perturbation of gene expression. Differentially expressed genes belonged to a range of metabolic pathways. A large proportion of regulated genes belonged to large gene families encoding exported proteins and surface antigens. The bulk regulation of genes belonging to large, co-expressed, multi-membered gene families has also been a feature of transcriptional responses of *Plasmodium* to a number of other stimuli (Clark et al. 2008; Fang et al. 2004) and is likely to represent a general rather than drug-specific response. We observed such a response in *Plasmodium* upon treatment of parasites with the apicoplast inhibitor thiostrepton (Tarr et al. 2011). Although the cause of this broadly observed phenomenon is unclear, given the co-expression of many functionally related genes during the *Plasmodium* lifecycle, a possible contribution to the differential expression of such genes comes from altered lifecycle progression in drug-treated or stressed parasites (Clark et al. 2008; Tarr et al. 2011). It is probable that this phenomenon contributes to the observed changes in expression of invasion-related genes and members of gene families in response to antimycin A. Similarly, we observed that while few genes encoding proteins involved in signalling processes were differentially expressed upon antimycin A treatment, increased expression of four genes coding for FIKK family kinases occurred i.e. 20% of this family. FIKK kinase genes are subtelomeric and their differential expression is in line with the differential expression of other multi-membered gene families.

Relatively few genes involved in stress response were differentially expressed in response to antimycin A treatment. This might suggest that the parasite responds to mtETC inhibition with limited upregulation (on a transcriptional level) of the cellular stress response. However, our data showed that stress response transcripts were almost exclusively upregulated upon antimycin A treatment. Furthermore, our functional enrichment analysis also indicated that genes within this category were enriched among upregulated transcripts. Indeed, it would not be unexpected that after 24 hours of treatment, the effects of antimycin A would be strongly felt beyond the mtETC Interestingly, the predominant downregulation of genes encoding transporters suggested that the parasite did not actively respond (on a transcriptional level) to remove the drug from the cell.

### Drug-specific Responses to Antimycin A

On the basis of the essential function of the *Plasmodium* mtETC, we hypothesised that *P. falciparum* may exhibit a gene expression response to antimycin A geared towards overcoming mitochondrial dysfunction, such as has been seen in yeast. Nineteen of the genes differentially expressed in response to antimycin A encoded proteins predicted to be targeted to the mitochondrion. The gene ontology enrichment analysis suggested an enrichment of proteins involved in respiration among the downregulated transcripts. Furthermore, a number of genes encoding mitochondrial chaperones were upregulated which may be indicative of a stress response directed at protecting mitochondrial proteins. Other upregulated genes encoding mitochondrially-targeted proteins were predominantly involved in protein import and folding. This functional distinction between up regulated and downregulated genes for mitochondrially-targeted proteins is of great interest given the target of antimycin A within the mitochondrion. Downregulated transcripts encoded proteins involved in electron transport and respiration, such as subunits of cytochrome *c* oxidase and complex III. These observations are in keeping with a somewhat typical retrograde signalling pathway. mtETC components are often targets of retrograde signalling in response to mitochondrial dysfunction (Scarpulla 2002). Additional mining of our dataset revealed downregulation of transcripts for subunits B, C and D of succinate dehydrogenase (known to be regulated on a transcriptional level by ‘Nuclear Respiratory Factors’ in other organisms (Scarpulla 2002)) upon antimycin A treatment in *P. falciparum*, albeit with p-values greater than 0.001. The gene encoding mtCpn10 (also known to be transcriptionally regulated in mammalian systems) was upregulated during the response to antimycin A of *P. falciparum*, and in addition, two genes for inner mitochondrial membrane translocase components were also upregulated. In mice, the gene encoding mtCpn10 contains recognition sites for Nuclear Respiratory Factor 1 (NRF-1) which regulates the expression of many nuclear encoded, mitochondrial genes involved in respiration (Scarpulla 2002).

The gene encoding cytochrome *c* exhibited upregulation by 2.2 fold (p = 0.0022). In other systems, the gene encoding cytochrome *c* is subject to regulation by NRF-1, as well as the protein being regulated post-translationally by phosphorylation (Hüttemann et al. 2007). Although there is no clear NRF-1 homologue in *Plasmodium*, the tyrosine residues involved in regulation of the protein in other organisms are conserved in the *Plasmodium* cytochrome *c* (data not shown). This, together with the upregulation of transcript levels in response to antimycin A, might suggest that this protein is important in the parasite's response to the drug, or, perhaps more generally, to mtETC dysfunction. For example, upregulation of the gene might allow increased removal of electrons from complex III under conditions of partial inhibition.

Given the close association of the apicoplast and mitochondrion, and in view of the possibility that the mitochondrion may depend on the plastid for translational precursors (Howe and Purton 2007), it was interesting to observe that only twenty-three differentially expressed genes (∼ 5%) encoded proteins predicted to be apicoplast-targeted.

The data presented here did not imply a strong response from genes involved in intracellular signalling, nor did this study address phosphorylation or allosteric regulation of mtETC components. However, were the observed transcriptional responses of genes encoding mtETC subunits reflected in the proteome of the mitochondrion, a protective response against antimycin A treatment could be envisaged; the downregulation of mtETC components upstream of complex III and upregulation of cytochrome *c* could act to reduce the production of (for example) superoxide. This is produced by the reaction of oxygen with semiquinone at the Q_o_ site of complex III upon inhibition by antimycin A at the Q_i_ site (Murphy 2009).

### Nucleotide Metabolism

Our gene ontology enrichment analysis revealed an enrichment of genes involved in nucleotide biosynthesis and transcription among upregulated transcripts. In particular, we observed the upregulation of four genes involved in pyrimidine metabolism. *Plasmodium* sp. must synthesise pyrimidines de novo (Gutteridge et al. 1979). Therefore, the gene expression responses observed here could indicate an attempt to increase general flux through the pyrimidine biosynthesis pathway in order to overcome inhibition of the pathway due to antimycin A treatment. Pyrimidine biosynthesis has already been suggested to be dependent on a functional mtETC (Painter et al. 2007). However, little is known about the fine transcriptional regulation of pyrimidine biosynthesis pathway components in *Plasmodium.*

In addition to the significant increases in expression of aspartate carbamoyl transferase and orotate phosphoribosyl transferase, transcripts for carbamoyl phosphate synthetase and dihydroorotase were also upregulated, albeit less significantly (2.13 fold, p = 0.0018 and 2.51 fold, p = 0.0013, respectively). These observations perhaps suggest co-regulation of these key genes in an attempt to increase flux through the inhibited pyrimidine biosythesis pathway (in this instance, due to antimycin A treatment). The strong 3.4 fold upregulation of the gene encoding orotate phosphoribosyl transferase was noteworthy given its observed upregulation in the presence of uridine in *Medicago truncatula* (Brady et al. 2010). The transcript-level regulation of these genes is in keeping with their regulation in other organisms. It should be noted that pyrimidine biosynthesis pathway enzymes are also regulated directly, and this study did not address the enzyme-level regulation of the pyrimidine biosynthesis pathway.

The effects of the expression of these genes require analysis at the protein level in order to characterise further this response. However, the observed changes in expression of genes encoding components of the pathway are in keeping with those seen in other organisms, giving further weight to the notion that pyrimidine biosynthesis is vital to *P. falciparum*.

The downregulation of genes involved in invasion also indicated an influence of expression timing on changes in expression in response to antimycin A, supporting a lifecycle or gene expression profile delay in antimycin A-treated parasites relative to controls. A lifecycle-dependent change in expression in response to antimycin A also goes some way to explaining the bulk regulation of co-expressed members of larger gene families. Slowing of lifecycle progression could indicate a general stress response due to perturbation of essential metabolism, or reflect an active delay in the cell cycle as the parasite ‘waits’ for (in this case) the drug pressure to be removed. Indeed, this would be in keeping with the effects of the antimalarial drug atovaquone on ring stage parasites (Painter et al. 2010). These observations may well be similar to the arrest/delay of ring stage parasites during 24 hours of atovaquone treatment (Painter et al. 2010). The gene expression data did not provide much indication of factors responsible for these effects. However, the majority of regulators involved in lifecycle progression of *P. falciparum* are just beginning to be understood (Koyama et al. 2009). Candidates highlighted in the microarray data presented here could include the genes encoding ApiAP2 proteins PF10_0075, PFL1075w and PF11_0091. While these require comprehensive characterisation, there is indication that one of the ApiAP2 domains of PF10_0075 has target sequence binding sites upstream of a number of developmental and invasion-related genes (Campbell et al. 2010). This raises the possibility that this protein may link the parasite's response to antimycin A with lifecycle progression.

The results presented here are in line with a ‘multi-level’ gene expression response to drug treatment. While downregulation of invasion-related genes is in keeping with an influence of delayed lifecycle progression on gene expression, the upregulation of enzymes of pyrimidine biosynthesis may be suggestive of an attempt to maintain the essential production of pyrimidines, which may link to the dependence of pyrimidine biosynthesis on the mtETC. Additionally, downregulation of genes encoding mtETC components combined with the increased expression of mitochondrial chaperones suggests a protective response against an inhibited mtETC, indicating regulation of nuclear genes encoding mitochondrion-targeted proteins upon disruption of mitochondrial function. The results presented here are suggestive of novel signalling pathways within *Plasmodium* and further investigation of the mechanisms underlying these responses will be crucial for understanding how the parasite counters drug challenges.

## Methods

***P. falciparum*** **culture**: Blood stage *P. falciparum* 3D7 was maintained in continuous culture in vitro at 37 °C using the candle-jar method (Trager and Jensen 1976). Cultures were maintained in citrated human blood at 2% hematocrit with complete culture media (RPMI 1640 + L-Glutamine (Gibco) with 5% w/v AlbuMAX I (Invitrogen), 50 μg ml^-1^ hypoxanthine, 25 mM HEPES (Gibco) and 25 μg ml^-1^ gentamycin (Sigma), pH7.6). Parasitaemia was assessed by thin blood smears stained with Giemsa (Sigma). Percentage infection of red blood cells was determined by cell counts under an oil immersion light microscope. Synchronous parasites were obtained by the Stockholm sorbitol method on two consecutive life cycles (Haeggström and Schlichtherle 2004). All work involving human blood was carried out in accordance with the Human Tissue Act (2004).

**Growth inhibition analysis:** The effect on growth of ranges of drug concentrations on *P. falciparum* cultures was assessed over one complete lifecycle. Experiments were conducted in triplicate 150 μl cultures in 96 well plates. Briefly, synchronous, ring stage parasites at 2% parasitaemia were treated 1:2000 with drug stocks at 2000 x concentration. Three to four drug concentrations were studied for each of four independent experiments. Cultures were incubated in the presence of the drug (or solvent in the case of controls) for 48 hours. Ring stage parasites present after 48 hours were deemed to have completed one lifecycle. Percent infection with ring stage parasites was ascertained by cell count as described above. Cell survival was expressed as a fraction of percent rings in treated cultures per percent rings in control cultures. Standard deviation was expressed as the combined standard deviation of percent parasitaemia in treated and untreated cultures.

**Gene expression analysis:** Triplicate 15 ml cultures containing synchronous, ring stage parasites at 5% parasitaemia were treated with 600 nM antimycin A in DMSO (or 1/2000 vol. DMSO for controls) for 24 hours. RNA was harvested using Trizol (Invitrogen) as described (Kyes 2004). RNA concentration and purity were determined using a NanoDrop (Thermo) and RNA integrity was tested using an Agilent Bioanalyzer (Cambridge Genomic Services, Department of Pathology).

Labelled RNA was hybridised to Plasmodium/Anopheles Genechip microarrays (Affymetrix). Microarray cRNA synthesis, hybridisation and plate-reading were conducted by Geneservice (Nottingham, UK). Raw CEL files were imported into Bioconductor (Gentleman et al. 2004) for data analysis. Microarrays were analysed for RNA degradation prior to normalisation. Non-*Plasmodium* probesets were removed using the altcdfenvs package (Gautier et al. 2004) and microarray data were normalised using GCRMA (Wu et al. 2004). The microarray data were deposited as a Gene Expression Omnibus accession (http://www.ncbi.nlm.nih.gov/geo/; GSE28625). Differential gene expression was analysed using Linear Models for Microarray Analysis (LIMMA) (Smyth 2004); normalised gene expression values for antimycin A-treated parasites were contrasted against untreated (DMSO) controls.

Gene annotations for the differentially expressed transcripts were downloaded from PlasmoDB (http://www.plasmodb.org). However, due to the gene-to-gene variation in annotation within PlasmoDB, categorisation of the differentially expressed transcripts was conducted manually, based the available gene ontology information.

Differential expression of twelve genes (PF14_0641, PF13_0040, PF14_0133, PFL1550w, PFI0735c, PF10_0334, MAL8P1.70, PF13_0058, MAL7P1.176, PF14_0373, MAL13P1.255 and PFF0160c) from the microarray analysis was validated by quantitative RT-PCR. Seryl-tRNA synthetase (PF07_0073) was used as an internal house-keeping control gene (Niang et al. 2009). Briefly, contaminating DNA was removed from extracted RNA using DNAfree (Ambion) as per manufacturer's instructions and cDNA was generated using Superscript II (Invitrogen) using 300 ng RNA randomly reverse primed with 100 ng random AT hexamer per 40 μl reaction, as per manufacturer's instructions (with the exception that reverse transcription was carried out at 37 °C). cDNA was diluted 1 in 5 for qRT-PCR analysis. qRT-PCR primers were designed using Primer3 (http://frodo.wi.mit.edu/primer3; see Supplementary Table 1 for primer sequences). Amplification efficiencies were confirmed prior to gene expression analysis. 5 μl of template cDNA was used per 25 μl qRT-PCR reaction, with 300 nM (final concentration) each forward and reverse primers in Sybr Green PCR premix (Applied Biosystems). qRT-PCR reactions were run in an ABIPrism 7300 real-time thermal cycler as appropriate for the Sybr Green PCR premix. Thermal cycling was followed by melt curve analysis. Baseline and threshold cycles were analysed using default settings. Relative fold changes in gene expression were calculated by the 2^-ΔΔCT^ relative quantification method (Livak and Schmittgen 2001). Reactions were conducted in technical quadruplicate and the raw C_T_ values of the replicates were subjected to the Grubb's test for outliers. The values were averaged and used in the subsequent fold-change calculation. The fold change values for three to four independent triplicate experiments were pooled for each gene and subjected to a non-parametric Mann-Whitney U test in StatView (version 4.51, Abacus Concepts). Correlation of qRT-PCR data with microarray-derived data was tested using the Spearman's Rank Correlation in StatView (Abacus Concepts, version 4.5).

**Gene ontology enrichment analysis:** Enrichment for biological terms in the lists of differentially expressed genes was analysed using the functional annotation clustering program of the Database for Annotation, Visualization and Integrated Discovery (Huang da et al. 2009a, b). The analysis was performed by inputting PlasmoDB gene identifiers for genes identified as being up- or downregulated by the microarray analysis, followed by comparison to all *P. falciparum* genes in order to assess enrichment. Analysis was performed under default conditions such that gene annotations for Clusters of Orthologous Groups ontology, Uniprot sequence features, biological process, cellular component and molecular function Gene Ontology terms, Kyoto Encyclopedia of Genes and Genomes pathways, Interpro domains, Protein Information Resource superfamily annotations and SMART domains were considered. Cluster names were derived from the biological terms found within each cluster. P-values outputted by DAVID were the geometric mean of the individual p-values (calculated by a Fisher's exact test) within each clustered group. Enrichment scores presented are the negative log of the p-values for each cluster.

## Figures and Tables

**Table 1:** DAVID functional clustering for upregulated or downreguated genes.

**DAVID Functional Clustering for upregulated genes**		
***Enrichment score***	***Description***	***p-value***
**2.37**	**Chaperones**	**4.27E-03**
**1.56**	**Nucleotide biosynthesis and transcription**	**2.75E-02**
**1.49**	**Stress response**	**3.24E-02**
**1.20**	**RNA helicase**	**6.31E-02**
0.95	Ligase	1.12E-01
0.86	Mitochondrial import	1.39E-01
0.86	Nucleotide binding	1.39E-01
0.85	Nucleotide metabolism	1.42E-01
0.76	Mitochondrial membrane	1.75E-01
0.50	tRNA metabolism	3.17E-01
0.46	rRNA metabolism	3.50E-01
0.43	Ion binding	3.72E-01
0.38	Translation	4.14E-01
0.32	RNA binding	4.84E-01
0.30	WD40 proteins	4.97E-01
0.29	Heat shock	5.13E-01
0.28	Protein complex assembly	5.28E-01
0.10	Proteolysis	7.90E-01

**DAVID Functional Clustering for downregulated genes**		
***Enrichment score***	***Description***	***p-value***
**3.20**	**Cytoskeleton binding**	**6.31E-04**
**2.44**	**Invasion**	**3.63E-03**
**1.55**	**Cytoskeleton organisation**	**2.82E-02**
**1.50**	**Ankyrin repeat proteins**	**3.16E-02**
**1.24**	**CoA metabolism and respiration**	**5.75E-02**
0.73	Metail ion transport	1.86E-01
0.73	Generation of mitochondrial metabolic precursors	1.87E-01
0.70	Transmembrane proteins	2.01E-01
0.66	Calcium binding	2.21E-01
0.54	Ion binding	2.90E-01
0.53	Inorganic cation transmembrane transport	2.97E-01
0.40	Protease	4.00E-01
0.33	Transcriptional regulation	4.69E-01
0.30	Kinases, nucleotide binding	5.05E-01
0.29	Non-membrane bound organelles	5.09E-01
0.28	Macromolecuar transport	5.20E-01
