# Supplementary material for: Transcript Level Responses of Plasmodium falciparum to Antimycin A
Source: Protist. 2012 Sep;163(5):755–66. doi: 10.1016/j.protis.2012.01.003 (PMC3657180; doi:10.1016/j.protis.2012.01.003)
Supplement: Supplementary file 1 [file mmc1.doc]

| Supplementary table 1  Sequences of qRT-PCR primers used for microarray validation, and associated melting temperatures | | |
| --- | --- | --- |
| Gene ID | Primer sequence | T_m_ ºC^a^ |
| PF14_0641 F | TCCATTGTCTCTGCTGGTTTCT | 64.9 |
| PF14_0641 R | GAAATGGACCTCCAGATGAACA | 65.0 |
| PF13_0040 F | CAACGTCAGAAGAGTTAGAAGAAACC | 64.1 |
| PF13_0040 R | GGTACCATTTCTCCTTCAGATGC | 64.7 |
| PF14_0133 F | GAAGCACACAATTATGGGAAGG | 64.3 |
| PF14_0133 R | ACCATAGGAAGTTCAACCGGATA | 64.3 |
| PFL1550w F | GAACGTGAACGAAGACAAGAAA | 63.1 |
| PFL1550w R | TCCATCAGATAAATTACCCATGC | 63.5 |
| PFI0735c F | CTACACCTACTGCACAAAATGCTAAA | 64.0 |
| PFI0735c R | AGAAGAGAATCTTCCTCCTTTTAATTC | 62.2 |
| PF10_0334 F | TTGTTTCCAACACGATCACATAC | 63.4 |
| PF10_0334 R | AAACGGGAGTCCAAATTCTTCT | 63.8 |
| PF14_0373 F | AACCGTCAAATCATCCACATC | 63.5 |
| PF14_0373 R | TTGGTTCAAATTTTCCAATTGAT | 62.9 |
| PFF0160c F | AAACAAATACCCATTATTGCATCA | 62.8 |
| PFF0160c R | CCAATGGCCTCCTTTAAATTG | 64.1 |
| MAL13P1.255 F | AACGGATATGTATGGGTTACCTAAAG | 62.8 |
| MAL13P1.255 R | GGTCGTGTATACGTATCTGGTGAA | 63.6 |
| MAL8P1.70 F | TGAAAATGCCACACAAAGGT | 63.0 |
| MAL8P1.70 R | GCTCTCGAATCGGATGTAAAA | 62.9 |
| PF13_0058 F | CCCTCAGCTTCTACCATAAATACC | 62.7 |
| PF13_0058 R | CCATGCCCATCCATCATTA | 64.2 |
| MAL7P1.176 F | GCATGGAGAATTTTTGCTTGA | 63.2 |
| MAL7P1.176 R | CGCATTTTCCTTTGGAACTT | 62.8 |
| PF07_0073 F * | GGGGCACATGGAAAGGATA | 64.6 |
| PF07_0073 R * | CTGCGTTGTTTAAAGCTCCTG | 63.7 |

* Internal housekeeping gene, seryl tRNA synthetase
