# Supplementary material for: Transcript Level Responses of Plasmodium falciparum to Antimycin A
Source: Protist. 2012 Sep;163(5):755–66. doi: 10.1016/j.protis.2012.01.003 (PMC3657180; doi:10.1016/j.protis.2012.01.003)
Supplement: Supplementary file 2 [file mmc2.doc]

| **Supplementary Table 2**  **Genes upregulated upon antimycin A treatment** | | | |
| --- | --- | --- | --- |
| Gene ID  (Probe set ID) | Description | Fold change | Adj.p.val |
| **Apicoplast targeted** | | | |
| PFD0075w (Pf.4.125.0_CDS_at) | Conserved Plasmodium falciparum protein family | 4.252 | 7.08E-04 |
| PFE0815w (Pf.5.208.0_CDS_at) | tRNA pseudouridine synthase, putative | 3.843 | 6.97E-04 |
| PF11_0324 (Pf.11.456.0_CDS_at) | Conserved Plasmodium protein, unknown function | 2.757 | 4.30E-04 |
| PF14_0439 (Pf.14.683.0_CDS_at) | M17 leucyl aminopeptidase | 2.015 | 8.25E-04 |
| PF13_0180 (Pf.13_1.220.0_CDS_at) | Cochaperonin | 1.932 | 6.49E-04 |
| PFB0270w (Pf.2.118.0_CDS_at) | Iron-sulfur assembly protein, putative | 1.866 | 8.86E-04 |
| PFI0680c (Pf.9.400.0_at) | Arginyl-tRNA synthetase, putative | 1.836 | 5.21E-04 |
| PFI0380c (Pf.9.163.0_CDS_at) | Formylmethionine deformylase, putative | 1.380 | 8.48E-04 |
| **Mitochondrion targeted** | | | |
| PF11_0265 (Pf.11.222.0_CDS_at) | Mitochondrial inner membrane translocase subunit TIM44, putative | 5.395 | 6.63E-05 |
| PFL0740c (Pf.12.453.0_CDS_at) | 10 kd chaperonin | 5.393 | 2.07E-04 |
| PF10_0144 (Pf.10.159.0_CDS_at) | Prohibitin, putative | 4.876 | 8.62E-05 |
| MAL7P1.150 (Pf.7.283.0_CDS_at) | Cysteine desulfurase, putative | 4.542 | 8.08E-04 |
| PFL1185c (Pf.12.475.0_CDS_at) | Cytochrome c heme lyase, putative | 4.250 | 7.06E-05 |
| PF13_0300 (Pf.13_1.280.0_CDS_at) | Mitochondrial inner membrane translocase, putative | 3.480 | 3.44E-04 |
| PF11_0188 (Pf.11.158.0_CDS_at) | Heat shock protein 90, putative | 3.302 | 1.87E-04 |
| PF11_0258 (Pf.11.447.0_CDS_at) | Co-chaperone GrpE, putative | 2.889 | 1.32E-04 |
| PF10_0153 (Pf.10.79.0_CDS_at) | Heat shock protein 60 | 2.710 | 5.04E-04 |
| PFF0205w (Pf.6.179.0_CDS_at) | Mitochondrial ribosomal protein L41 precursor, putative | 2.424 | 3.58E-04 |
| PFL0415w (Pf.12.91.0_CDS_at) | Mitochondrial ACP precursor | 2.361 | 4.61E-04 |
| PFB0545c (Pf.2.65.0_CDS_at) | Mitochondrial ribosomal protein L12 precursor, putative | 2.059 | 2.34E-04 |
| PFF0155w (Pf.6.43.0_CDS_at) | Bcs1 protein, putative | 1.921 | 9.08E-05 |
| PFD0600c (Pf.4.106.0_CDS_at) | Mitochondrial ribosomal protein S12 precursor, putative | 1.495 | 7.03E-04 |
| **Motor proteins and cytoskeletal proteins** | | | |
| PFF1480w (Pf.6.235.0_CDS_at) | Microtubule-associated protein ytm1 homologue, putative | 6.177 | 1.18E-04 |
| PF14_0124 (Pf.14.97.0_CDS_a_at) | Actin II | 5.705 | 9.31E-04 |
| PFL1475w (Pf.12.355.0_CDS_at) | Sun-family protein, putative | 2.145 | 3.71E-04 |
| **Kinases** | | | |
| MAL7P1.144 (Pf.7.59.0_CDS_at) | Serine/Threonine protein kinase, FIKK family | 9.105 | 8.53E-04 |
| PF10_0380 (Pf.10.178.0_CDS_at) | Serine/Threonine protein kinase, FIKK family | 8.233 | 5.13E-04 |
| MAL13P1.185 (Pf.13_1.319.0_CDS_a_at) | Protein kinase 6 | 3.914 | 9.08E-05 |
| PFD0755c (Pf.4.109.0_CDS_at) | Adenylate kinase 1 | 3.186 | 3.86E-04 |
| MAL7P1.175 (Pf.7.220.0_CDS_at) | Serine/Threonine protein kinase, FIKK family, pseudogene | 2.559 | 7.24E-04 |
| PFL0040c (Pf.12.420.0_CDS_at) | Serine/Threonine protein kinase, FIKK family | 2.320 | 8.41E-04 |
| **Phosphatases** | | | |
| PFE1035c (Pf.5.115.0_CDS_at) | BIS(5'-nucleosyl)-tetraphosphatase (Diadenosine tetraphosphatase), putative | 3.306 | 5.74E-04 |
| PF14_0022 (Pf.14.60.1_CDS_a_at) | Exopolyphosphatase, putative | 2.511 | 2.50E-04 |
| PF07_0110 (Pf.7.206.0_CDS_at) | Protein phosphatase, putative | 2.111 | 7.34E-04 |
| MAL13P1.275 (Pf.13_1.279.0_CDS_at) | Protein phosphatase, putative | 1.802 | 7.34E-04 |
| **Transporter** | | | |
| PF14_0662 (Pf.14.531.0_CDS_at) | Nucleoside transporter, putative | 2.384 | 3.50E-04 |
| **Heat shock, chaperone or stress response proteins** | | | |
| PFB0090c (Pf.2.173.0_CDS_at) | RESA-like protein with PHIST and DnaJ domains | 7.756 | 3.55E-04 |
| PFB0635w (Pf.2.129.0_CDS_at) | T-complex protein 1, putative | 3.272 | 5.31E-04 |
| PF14_0576 (Pf.14.503.0_CDS_a_at) | Ubiquitin carboxyl-terminal hydrolase, putative | 3.208 | 8.94E-04 |
| PFE0605c (Pf.5.281.0_CDS_at) | Glutathione synthetase | 3.180 | 7.22E-04 |
| PF11_0351 (Pf.11.66.0_CDS_at) | Heat shock protein hsp70 homologue | 3.060 | 9.40E-04 |
| PFC0360w (Pf.3.54.0_CDS_at) | Activator of Hsp90 ATPase homolog 1-like protein, putative | 2.921 | 4.72E-04 |
| PFA0660w (Pf.1.99.0_CDS_at) | Protein with DNAJ domain, dnj1/sis1 family | 2.787 | 9.31E-04 |
| PF14_0324 (Pf.14.209.0_CDS_at) | Hsp70/Hsp90 organizing protein, putative | 2.399 | 4.42E-04 |
| MAL13P1.283 (Pf.13_1.331.0_CDS_at) | TCP-1/cpn60 chaperonin family, putative | 2.313 | 4.28E-04 |
| PF11_0331 (Pf.11.232.0_CDS_at) | TCP-1/cpn60 chaperonin family | 2.188 | 5.30E-04 |
| PFL2175w (Pf.12.383.0_CDS_at) | Ubiquitin conjugating enzyme E2, putative | 2.103 | 6.13E-04 |
| PF13_0021 (Pf.13_1.245.0_CDS_at) | Small heat shock protein, putative | 1.991 | 7.24E-04 |
| PFI1030c (Pf.9.105.0_CDS_at) | Ubiquitin conjugating enzyme, putative | 1.983 | 2.05E-04 |
| PFC0271c (Pf.3.65.0_CDS_at) | Glutaredoxin 1 | 1.971 | 2.21E-04 |
| PFF0340c (Pf.6.56.0_CDS_at) | Glutaredoxin-like protein, putative | 1.952 | 2.06E-04 |
| PFF0430w (Pf.6.189.0_CDS_at) | Chaperone, putative | 1.805 | 3.86E-04 |
| PF11_0216 (Pf.11.101.1_CDS_a_at) | Heat shock factor binding protein 1, putative | 1.713 | 3.36E-04 |
| PFC0365w (Pf.3.23.0_CDS_at) | PRP19-like protein, putative | 1.598 | 3.93E-04 |
| PFI1250w (Pf.9.247.0_CDS_at) | Thioredoxin-like protein 2 | 1.328 | 1.52E-04 |
| **Transcription, translation or nucleotide binding** | | | |
| PFD0565c (Pf.4.105.0_CDS_at) | DEAD box ATP-dependent RNA helicase, putative | 4.513 | 7.53E-04 |
| PF11_0471 (Pf.11.482.0_CDS_at) | Nucleolar preribosomal assembly protein, putative | 3.858 | 3.14E-04 |
| PFL0330c (Pf.12.433.0_CDS_at) | DNA-directed RNA polymerase III subunit, putative | 3.497 | 5.46E-04 |
| PF14_0413 (Pf.14.237.0_CDS_at) | CAF1 family ribonuclease, putative | 3.465 | 9.69E-04 |
| PFF0100w (Pf.6.175.0_CDS_at) | ATP-dependent RNA Helicase, putative | 3.382 | 5.08E-04 |
| PF11_0305 (Pf.11.160.0_CDS_at) | RNA methyltransferase, putative | 3.346 | 1.50E-04 |
| PFE0800w (Pf.5.128.0_CDS_at) | RAP protein, putative | 3.331 | 3.49E-04 |
| PF07_0122 (Pf.7.124.0_CDS_at) | Nucleolus BRIX protein, putative | 3.112 | 9.31E-04 |
| PF13_0286 (Pf.13_1.645.0_CDS_at) | Methyltransferase, putative | 3.074 | 4.61E-04 |
| PFF1500c (Pf.6.316.0_CDS_at) | DEAD/DEAH box ATP-dependent RNA helicase, putative | 2.990 | 1.67E-04 |
| PFL2475w (Pf.12.240.0_CDS_x_at) | DEAD/DEAH box helicase, putative | 2.962 | 3.81E-04 |
| PF10_0300 (Pf.10.274.0_CDS_at) | RNA methyltransferase, putative | 2.902 | 9.17E-05 |
| PF13_0177 (Pf.13_1.367.0_CDS_at) | ATP-dependent RNA Helicase, putative | 2.867 | 1.87E-04 |
| PFE0465c (Pf.5.101.0_CDS_at) | RNA polymerase I | 2.843 | 9.08E-05 |
| PF11_0358 (Pf.11.460.0_CDS_at) | DNA-directed RNA polymerase 1, subunit 2, putative | 2.839 | 3.50E-04 |
| MAL13P1.341 (Pf.13_1.673.0_CDS_at) | Ribosome biogenesis protein MRT4, putative | 2.710 | 2.87E-04 |
| PF07_0027 (Pf.7.131.0_CDS_at) | DNA-directed RNA polymerase 2 8.2 kDa polypeptide, putative | 2.601 | 2.40E-04 |
| PF11_0071 (Pf.11.404.0_CDS_at) | RuvB DNA helicase, putative | 2.596 | 3.67E-04 |
| PF07_0071 (Pf.7.182.0_CDS_at) | Queuine tRNA-ribosyltransferase, putative | 2.457 | 4.96E-04 |
| PFL0665c (Pf.12.447.0_CDS_at) | RNA polymerase subunit 8c, putative | 2.367 | 9.31E-04 |
| PF11_0454 (Pf.11.148.0_CDS_at) | 40S ribosomal protein S21e, putative | 2.284 | 6.53E-04 |
| PF14_0028 (Pf.14.302.0_CDS_at) | Pre-mRNA splicing factor, putative | 2.278 | 9.31E-04 |
| PF14_0174 (Pf.14.171.0_CDS_at) | Pseudouridine synthase, putative | 2.268 | 7.03E-04 |
| PF14_0207 (Pf.14.637.0_CDS_x_at) | RNA polymerase subunit, putative | 2.216 | 3.52E-04 |
| PF14_0198 (Pf.14.412.0_CDS_at) | Glycine-tRNA ligase, putative | 2.192 | 3.71E-04 |
| PFA0570w (Pf.1.44.0_CDS_at) | Conserved Plasmodium protein, unknown function | 2.176 | 4.36E-04 |
| PF14_0589 (Pf.14.508.0_CDS_at) | Valine-tRNA ligase, putative | 2.098 | 3.55E-04 |
| PF14_0072 (Pf.14.379.0_CDS_at) | Nucleolar preribosomal GTPase, putative | 2.096 | 6.49E-04 |
| PFL2010c (Pf.12.191.0_CDS_at) | DEAD/DEAH box helicase, putative | 2.018 | 8.47E-04 |
| PF13_0313 (Pf.13_1.518.0_CDS_at) | Zinc finger protein, putative | 1.965 | 4.54E-04 |
| PFL2150c (Pf.12.278.0_CDS_at) | CCCH-type Zn-finger protein, putative | 1.960 | 3.39E-04 |
| PF14_0784 (Pf.14.776.0_at) | Ribosome biogenesis protein, NOP10-like | 1.931 | 3.14E-04 |
| PF10_0187 (Pf.10.252.0_CDS_at) | 60S ribosomal protein L30e, putative | 1.925 | 5.66E-04 |
| PF08_0037 (Pf.8.107.0_CDS_at) | RNA polymerase II mediator complex protein MED7, putative | 1.924 | 4.36E-04 |
| PF14_0620 (Pf.14.288.0_CDS_at) | Metal-dependent hydrolase, putative | 1.916 | 8.33E-04 |
| PF11_0191 (Pf.11.132.0_CDS_at) | Pre-RNA processing ribonucleoprotein, putative | 1.854 | 2.40E-04 |
| PFF1400w (Pf.6.93.0_CDS_at) | RAP protein, putative | 1.851 | 7.47E-04 |
| PF13_0037 (Pf.13_1.291.0_CDS_at) | DEAD box helicase, putative | 1.812 | 4.72E-04 |
| PF10_0041 (Pf.10.328.0_CDS_at) | U5 small nuclear ribonuclear protein, putative | 1.788 | 9.27E-04 |
| PFF0345w (Pf.6.26.0_CDS_at) | Translation initiation factor IF-2, putative | 1.781 | 6.24E-04 |
| PF13_0257 (Pf.13_1.637.0_CDS_at) | Glutamate--tRNA ligase, putative | 1.775 | 6.04E-04 |
| PFI0190w (Pf.9.196.0_CDS_at) | 60S ribosomal protein L32, putative | 1.718 | 6.33E-04 |
| PF13_0214 (Pf.13_1.45.0_CDS_at) | Elongation factor 1-gamma, putative | 1.696 | 1.65E-04 |
| PF10_0194 (Pf.10.199.0_CDS_at) | NOP12-like protein | 1.656 | 7.83E-04 |
| PF10_0075 (Pf.10.131.0_CDS_x_at) | Transcription factor with AP2 domain(s), putative | 1.606 | 8.33E-04 |
| PF13_0214 (Pf.13_1.45.1_a_at) | Elongation factor 1-gamma, putative | 1.599 | 8.48E-04 |
| PFL1075w (Pf.12.339.0_CDS_at) | Transcription factor with AP2 domain(s), putative | 1.585 | 3.84E-04 |
| PF07_0043 (Pf.7.245.0_CDS_at) | 60S ribosomal protein L34-A, putative | 1.553 | 8.23E-04 |
| PF11_0065 (Pf.11.401.0_CDS_at) | 40S ribosomal protein S4, putative | 1.455 | 8.59E-04 |
| PF10_0291 (Pf.10.378.0_CDS_at) | RAP protein, putative | 1.433 | 4.11E-04 |
| PFL1170w (Pf.12.148.0_CDS_at) | Polyadenylate-binding protein, putative | 1.305 | 5.21E-04 |
| **Exported, membrane or surface proteins** | | | |
| PFD1140w (Pf.4.167.0_CDS_at) | Plasmodium exported protein (PHISTc), unknown function | 8.794 | 1.71E-04 |
| PFB0953w (Pf.2.145.0_CDS_at) | Plasmodium exported protein (hyp15), unknown function | 8.643 | 6.86E-05 |
| PF14_0746 (Pf.14.563.0_CDS_at) | Plasmodium exported protein (PHISTb), unknown function | 8.460 | 6.15E-04 |
| PF11_0511 (Pf.11.491.0_CDS_at) | Plasmodium exported protein, unknown function | 7.337 | 2.08E-04 |
| PFI1750c (Pf.9.360.0_CDS_at) | Plasmodium exported protein (hyp11), unknown function | 7.095 | 1.89E-04 |
| PFI1770w (Pf.9.156.0_CDS_at) | Plasmodium exported protein (PHISTb), unknown function | 7.088 | 5.61E-05 |
| PF10_0006 (Pf.10.315.0_CDS_at) | Rifin | 6.360 | 7.37E-04 |
| MAL13P1.480 (X03144.1_at) | Histidine-rich protein III | 5.261 | 3.15E-04 |
| PF07_0008 (Pf.7.87.0_CDS_at) | Plasmodium exported protein, unknown function | 4.936 | 6.25E-04 |
| PFB0080c (Pf.2.171.0_CDS_at) | Plasmodium exported protein (PHISTb), unknown function | 4.864 | 6.89E-04 |
| PF11_0506 (Pf.11.369.0_CDS_at) | Antigen 332, DBL-like protein | 4.709 | 2.05E-04 |
| MAL8P1.3 (Pf.8.231.0_CDS_at) | Plasmodium exported protein (hyp9), unknown function | 4.473 | 1.69E-04 |
| PFE1600w (Pf.5.94.0_CDS_at) | Plasmodium exported protein (PHISTb), unknown function | 3.878 | 2.40E-04 |
| PF11_0037 (Pf.11.85.0_CDS_at) | Plasmodium exported protein (PHISTb), unknown function | 3.607 | 9.31E-04 |
| PFL1800w (Pf.12.221.0_CDS_at) | Conserved Plasmodium protein, unknown function | 3.560 | 3.52E-04 |
| PF13_0275 (AJ290931.1_RC_s_at) | Plasmodium exported protein, unknown function | 3.393 | 2.50E-04 |
| PF14_0536 (Pf.14.708.0_CDS_at) | Conserved Plasmodium protein, unknown function | 3.306 | 3.91E-04 |
| PF08_0137 (Pf.8.222.0_CDS_at) | Plasmodium exported protein (PHISTc), unknown function | 3.063 | 8.52E-04 |
| PF10_0215 (Pf.10.143.0_CDS_at) | Conserved Plasmodium membrane protein, unknown function | 3.019 | 9.31E-04 |
| PFI1760w (Pf.9.269.0_CDS_at) | Ring-exported protein 4 | 2.992 | 9.88E-04 |
| PFI1040c (Pf.9.337.0_CDS_at) | Conserved Plasmodium membrane protein, unknown function | 2.962 | 3.57E-04 |
| PFE0245c (Pf.5.270.0_CDS_at) | Conserved Plasmodium membrane protein, unknown function | 2.722 | 5.36E-04 |
| MAL7P1.15 (Pf.7.129.0_CDS_at) | Conserved Plasmodium membrane protein, unknown function | 2.675 | 8.08E-04 |
| PF14_0014 (Pf.14.368.0_CDS_at) | Plasmodium exported protein, unknown function | 2.584 | 9.17E-05 |
| PFA0055c (Pf.1.116.0_CDS_s_at) | Plasmodium exported protein (hyp5), unknown function | 2.454 | 1.59E-04 |
| PFF0850c (Pf.6.288.0_CDS_at) | Stevor | 2.443 | 5.95E-04 |
| PFE1590w (Pf.5.40.0_CDS_at) | Early transcribed membrane protein 5, ETRAMP5 | 2.392 | 2.15E-04 |
| PFB0100c (Pf.2.8.0_CDS_s_at) | Knob-associated histidine-rich protein | 2.343 | 5.02E-04 |
| PFB0921c (Pf.2.11.0_CDS_a_at) | Plasmodium exported protein, unknown function | 2.205 | 4.78E-04 |
| PFB0930w (Pf.2.87.0_CDS_at) | Plasmodium exported protein (hyp9), unknown function | 2.091 | 1.42E-04 |
| MAL7P1.31 (Pf.7.244.0_CDS_at) | Conserved Plasmodium membrane protein, unknown function | 2.082 | 4.73E-04 |
| PF11_0224 (Pf.11.12.0_CDS_at) | Circumsporozoite-related antigen | 1.877 | 7.36E-04 |
| PFE0755c (Pf.5.289.0_CDS_at) | Rhomboid protease ROM9 | 1.865 | 7.72E-04 |
| PFL1065c (Pf.12.472.0_CDS_at) | Conserved Plasmodium protein, unknown function | 1.545 | 6.90E-04 |
| PF14_0532 (Pf.14.491.0_CDS_at) | LCCL domain-containing protein CCP2 | 1.455 | 4.18E-04 |
| MAL7P1.12 (Pf.7.27.0_CDS_s_at) | Erythrocyte membrane-associated antigen | 1.365 | 7.83E-04 |
| **Other metabolism** | | | |
| MAL13P1.284 (Pf.13_1.236.0_CDS_at) | Pyrroline carboxylate reductase | 5.577 | 1.69E-04 |
| PF11_0165 (Pf.11.111.0_CDS_s_at) | Falcipain-2A | 5.417 | 8.08E-04 |
| PFI1020c (Pf.9.104.0_CDS_a_at) | Inosine-5'-monophosphate dehydrogenase | 5.264 | 1.86E-04 |
| PF08_0006 (Pf.8.125.0_CDS_at) | Prohibitin, putative | 5.230 | 8.62E-05 |
| PF14_0100 (Pf.14.385.0_CDS_at) | Cytidine triphosphate synthetase | 5.019 | 9.69E-04 |
| PFI1140w (Pf.9.240.0_CDS_at) | Flavodoxin-like protein | 4.628 | 4.42E-04 |
| PF13_0144 (Pf.13_1.362.0_CDS_at) | Oxidoreductase, putative | 4.520 | 7.08E-04 |
| PFF1025c (Pf.6.106.0_CDS_at) | Pyridoxine/pyridoxal 5-phosphate biosynthesis enzyme | 4.256 | 3.44E-04 |
| PF14_0534 (Pf.14.707.0_CDS_at) | Serine hydroxymethyltransferase, putative | 4.155 | 7.83E-04 |
| PF13_0259 (Pf.13_1.496.0_CDS_at) | Cytidine and deoxycytidylate deaminase, putative | 3.986 | 6.18E-05 |
| PF10_0150 (Pf.10.120.0_CDS_at) | Methionine aminopeptidase, putative | 3.650 | 7.05E-04 |
| PFE0630c (Pf.5.105.0_CDS_at) | Orotate phosphoribosyltransferase | 3.420 | 3.05E-04 |
| PFF1360w (Pf.6.228.0_CDS_at) | 6-pyruvoyltetrahydropterin synthase | 3.184 | 6.78E-04 |
| MAL13P1.184 (Pf.13_1.371.0_CDS_at) | Endopeptidase, putative | 2.979 | 3.41E-04 |
| PFF1335c (Pf.6.39.0_CDS_at) | 4-methyl-5(B-hydroxyethyl)-thiazol monophosphate biosynthesis enzyme | 2.790 | 6.60E-05 |
| PFL1845c (Pf.12.505.0_CDS_at) | Calcyclin binding protein, putative | 2.554 | 4.72E-04 |
| PF10_0058 (Pf.10.185.0_CDS_at) | DNAJ protein, putative | 2.534 | 8.08E-04 |
| PFE1240w (Pf.5.230.0_CDS_at) | Conserved protein, unknown function | 2.531 | 9.31E-04 |
| PFF0580w (Pf.6.21.0_CDS_at) | Lsm12, putative | 2.465 | 9.50E-04 |
| PF13_0157 (Pf.13_1.262.0_CDS_at) | Ribose-phosphate pyrophosphokinase, putative | 2.424 | 2.05E-04 |
| PFE1115c (Pf.5.306.0_CDS_at) | S-adenosylmethionine-dependent methyltransferase, putative | 2.421 | 9.27E-04 |
| MAL13P1.221 (Pf.13_1.165.0_CDS_at) | Aspartate carbamoyltransferase | 2.366 | 4.81E-04 |
| PFB0280w (Pf.2.120.0_CDS_at) | EPSP-SK, putative | 2.324 | 9.08E-05 |
| PF11_0250 (Pf.11.134.0_CDS_at) | High mobility group-like protein NHP2, putative | 2.315 | 6.60E-05 |
| PFL2050w (Pf.12.380.0_CDS_at) | Protein geranylgeranyltransferase type II, alpha subunit, putative | 2.177 | 8.47E-04 |
| PF14_0242 (Pf.14.231.0_CDS_at) | Arginine-N-methyltransferase, putative | 2.075 | 5.29E-04 |
| PF07_0033 (Pf.7.18.0_CDS_at) | Cg4 protein | 1.905 | 3.52E-04 |
| PF14_0378 (Pf.14.8.0_CDS_at) | Triosephosphate isomerase | 1.850 | 6.47E-04 |
| MAL13P1.294 (Pf.13_1.520.0_CDS_at) | GTP binding protein, putative | 1.849 | 7.47E-04 |
| PFI1570c (Pf.9.85.0_CDS_at) | M18 aspartyl aminopeptidase | 1.815 | 6.43E-04 |
| PF14_0570 (Pf.14.716.0_CDS_at) | Pyridoxal 5'-phosphate synthase, putative | 1.765 | 2.65E-04 |
| PFA0520c (Pf.1.57.0_CDS_at) | Chromatin assembly factor 1 protein WD40 domain, putative | 1.762 | 8.59E-04 |
| PF13_0287 (Pf.13_1.102.0_CDS_a_at) | Adenylosuccinate synthetase | 1.725 | 7.72E-04 |
| PFA0355w (Pf.1.80.0_CDS_at) | Carbon catabolite repressor protein 4, putative | 1.695 | 5.88E-04 |
| PFA0465c (Pf.1.137.0_CDS_at) | N-terminal acetyltransferase, putative | 1.623 | 9.50E-04 |
| PFL1420w (Pf.12.9.0_CDS_a_at) | Macrophage migration inhibitory factor homologue | 1.398 | 3.52E-04 |
| PF10_0328 (Pf.10.74.0_CDS_at) | Bromodomain protein, putative | 1.186 | 4.67E-04 |
