# Supplementary material for: Transcript Level Responses of Plasmodium falciparum to Antimycin A
Source: Protist. 2012 Sep;163(5):755–66. doi: 10.1016/j.protis.2012.01.003 (PMC3657180; doi:10.1016/j.protis.2012.01.003)
Supplement: Supplementary file 3 [file mmc3.doc]

| Supplementary Table 3  **Genes downregulated upon antimycin A treatment** | | | |
| --- | --- | --- | --- |
| Gene ID  (Probeset ID) | Product Description | Fold change | Adj.p.val |
| **Apicoplast targeted** | | | |
| PF10_0138 (Pf.10.242.0_CDS_at) | Conserved Plasmodium protein, unknown function | 0.079 | 7.06E-05 |
| MAL13P1.49 (Pf.13_1.418.0_CDS_a_at) | Conserved Plasmodium protein, unknown function | 0.100 | 1.18E-04 |
| PFL0035c (J04007.1_s_at) | Acyl-CoA synthetase, PfACS7 | 0.102 | 2.77E-04 |
| PF10_0119 (Pf.10.17.0_CDS_at) | Conserved Plasmodium protein, unknown function | 0.118 | 9.08E-05 |
| PFD0230c (Pf.4.39.0_CDS_at) | Protease, putative | 0.135 | 6.05E-04 |
| MAL8P1.73 (Pf.8.46.0_CDS_at) | Conserved Plasmodium protein, unknown function | 0.141 | 2.88E-04 |
| PF11_0304 (Pf.11.120.0_CDS_at) | Conserved Plasmodium protein, unknown function | 0.146 | 2.15E-04 |
| MAL13P1.114 (Pf.13_1.72.0_CDS_at) | Conserved Plasmodium protein, unknown function | 0.159 | 1.34E-04 |
| PFB0570w (Pf.2.80.0_CDS_at) | SPATR protein, putative | 0.184 | 9.08E-05 |
| MAL13P1.297 (Pf.13_1.521.0_CDS_at) | ADP-ribosylation factor, putative | 0.271 | 8.74E-04 |
| PFF1420w (Pf.6.231.0_CDS_at) | Phosphatidylcholine-sterol acyltransferase precursor, putative | 0.272 | 3.84E-04 |
| PFL0840c (Pf.12.175.0_CDS_at) | Conserved Plasmodium protein, unknown function | 0.321 | 4.36E-04 |
| PFF0595c (Pf.6.15.0_CDS_at) | Leucine-rich repeat protein 5, LRR5 | 0.354 | 3.84E-04 |
| PFL2330w (Pf.12.238.0_CDS_at) | Conserved Plasmodium protein, unknown function | 0.574 | 4.02E-04 |
| **Mitochondrion-targeted** | | | |
| PFI1375w (Pf.9.52.0_CDS_at) | Cytochrome c oxidase, putative | 0.470 | 7.24E-04 |
| PF13_0327 (Pf.13_1.78.0_CDS_at) | Cytochrome c oxidase subunit 2, putative | 0.478 | 1.66E-04 |
| PF10_0218 (Pf.10.354.0_CDS_at) | Citrate synthase, mitochondrial precursor, putative | 0.482 | 3.92E-04 |
| PF10_0120 (Pf.10.137.0_CDS_at) | Ubiquinol-cytochrome c reductase complex subunit, putative | 0.556 | 2.05E-04 |
| PFL0110c (Pf.12.82.0_CDS_at) | Mitochondrial phosphate carrier protein | 0.596 | 3.50E-04 |
| **Motor proteins and cytoskeletal proteins** | | | |
| PFL2460w (Pf.12.81.0_CDS_at) | Coronin | 0.045 | 5.55E-06 |
| PFF0675c (Pf.6.149.0_CDS_at) | Myosin E | 0.076 | 6.12E-05 |
| PFL1435c (Pf.12.126.0_CDS_at) | Myosin D | 0.118 | 4.05E-04 |
| PFE1545c (Pf.5.324.0_CDS_at) | Formin 1, putative | 0.157 | 6.52E-04 |
| PF13_0326 (Pf.13_1.283.0_CDS_at) | Actin-depolymerizing factor, putative | 0.224 | 7.65E-04 |
| MAL8P1.146 (Pf.8.10.0_CDS_at) | Filament assembling protein, putative | 0.239 | 1.42E-04 |
| PFA0260c (Pf.1.15.0_CDS_at) | Adenylate cyclase-associated protein, putative | 0.284 | 7.19E-04 |
| MAL7P1.137 (Pf.7.144.0_CDS_at) | Kelch protein, putative | 0.514 | 5.17E-04 |
| PFA0190c (Pf.1.128.0_CDS_at) | Actin-related protein, ARP1 | 0.602 | 2.50E-04 |
| **Invasion-related proteins** | | | |
| PFL2505c (Pf.12.198.0_CDS_at) | Rhoptry neck protein 3, putative | 0.087 | 4.55E-04 |
| PF14_0495 (Pf.14.478.0_CDS_at) | Rhoptry neck protein 2 | 0.088 | 3.23E-05 |
| PF13_0233 (Pf.13_1.99.0_CDS_at) | Myosin A | 0.095 | 7.43E-04 |
| PF11_0344 (AF352829.1_s_at) | Apical membrane antigen 1, AMA1 | 0.097 | 4.81E-05 |
| PF10_0039 (Pf.10.51.0_CDS_at) | Membrane skeletal protein IMC1-related | 0.097 | 3.97E-04 |
| PFE1285w (Pf.5.22.0_CDS_at) | Membrane skeletal protein IMC1-related | 0.103 | 2.50E-04 |
| PFC0185w (Pf.3.13.0_CDS_at) | Membrane skeletal protein IMC1-related | 0.103 | 4.72E-04 |
| PF11_0168 (Pf.11.16.0_CDS_at) | Moving junction protein | 0.105 | 1.37E-04 |
| PFE0075c (Pf.5.41.0_CDS_at) | Rhoptry-associated protein 3, RAP3 | 0.105 | 2.40E-04 |
| PFB0680w (Pf.2.44.0_CDS_at) | Rhoptry neck protein 6 | 0.111 | 2.72E-05 |
| PF14_0102 (Pf.14.2.0_CDS_at) | Rhoptry-associated protein 1, RAP1 | 0.142 | 1.59E-04 |
| PFL2225w (Pf.12.570.0_at) | Myosin A tail domain interacting protein | 0.146 | 5.60E-05 |
| PFE0080c (Pf.5.9.0_CDS_at) | Rhoptry-associated protein 2, RAP2 | 0.179 | 3.71E-04 |
| PFI0265c (M17530.1_s_at) | RhopH3 | 0.208 | 2.05E-04 |
| PFL1090w (Pf.12.145.0_CDS_at) | Glideosome-associated protein 45 | 0.247 | 2.06E-04 |
| PFI1445w (Pf.9.5.0_CDS_a_at) | High molecular weight rhoptry protein-2 | 0.288 | 6.30E-04 |
| PFI0880c (Pf.9.7.0_CDS_at) | Glideosome-associated protein 50 | 0.432 | 6.60E-05 |
| **Kinases** | | | |
| PFB0665w (Pf.2.131.0_CDS_at) | Serine/threonine protein kinase, putative | 0.039 | 7.30E-05 |
| PF11_0464 (Pf.11.2.0_CDS_at) | Ser/Thr protein kinase, putative | 0.109 | 9.23E-05 |
| PFB0815w (Pf.2.13.0_CDS_at) | Calcium-dependent protein kinase 1 | 0.118 | 2.40E-04 |
| PF14_0346 (Pf.14.330.0_CDS_at) | cGMP-dependent protein kinase | 0.143 | 1.23E-04 |
| PFI1685w (Pf.9.55.0_CDS_at) | cAMP-dependent protein kinase catalytic subunit | 0.197 | 1.42E-04 |
| MAL7P1.18 (Pf.7.13.0_CDS_at) | Serine/threonine protein kinase, putative | 0.199 | 7.08E-04 |
| PF11_0362 (Pf.11.235.0_CDS_at) | Protein phosphatase, putative | 0.260 | 2.05E-04 |
| PFL1885c (Pf.12.508.0_CDS_at) | Calcium/calmodulin-dependent protein kinase 2 | 0.265 | 7.06E-05 |
| PF13_0166 (Pf.13_1.366.0_CDS_at) | Protein kinase, putative | 0.407 | 3.71E-04 |
| PF11_0060 (Pf.11.125.0_CDS_at) | Calcium/calmodulin-dependent protein kinase, putative | 0.435 | 9.45E-04 |
| **Phosphatases** | | | |
| PF14_0224 (Pf.14.420.1_CDS_a_at) | Serine/threonine protein phosphatase | 0.064 | 3.23E-05 |
| PFL0300c (Pf.12.246.0_CDS_at) | Protein phosphatase, putative | 0.099 | 8.99E-04 |
| PF10_0177a (Pf.10.25.0_CDS_at) | Serine/threonine protein phosphatase, putative | 0.152 | 3.80E-04 |
| PF14_0492 (Pf.14.33.0_CDS_a_at) | Protein phosphatase 2b regulatory subunit, putative | 0.431 | 6.25E-04 |
| PF14_0142 (Pf.14.194.0_CDS_at) | Serine/threonine protein phosphatase | 0.491 | 7.63E-04 |
| PFC0710w-a (Pf.3.2.0_CDS_s_at) | Inorganic pyrophosphatase, putative | 0.662 | 6.49E-04 |
| **Transporters** | | | |
| PFL1700c (Pf.12.270.0_CDS_at) | V-type K+-independent H+-translocating inorganic pyrophosphatase | 0.091 | 9.69E-04 |
| MAL8P1.13 (Pf.8.101.0_CDS_at) | Folate/biopterin transporter, putative | 0.115 | 7.34E-04 |
| PFB0275w  (Pf.2.259.0_at) | Metabolite/drug transporter, putative | 0.143 | 3.41E-04 |
| PFB0435c (Pf.2.190.0_CDS_at) | Transporter, putative | 0.224 | 1.93E-04 |
| PFL2220w (Pf.12.232.0_CDS_at) | Conserved Plasmodium protein, unknown function | 0.237 | 2.14E-04 |
| PFE0785c (Pf.5.108.0_CDS_at) | Metabolite/drug transporter, putative | 0.242 | 2.77E-04 |
| PF14_0369 (Pf.14.451.0_CDS_at) | Copper transporter putative | 0.353 | 7.23E-04 |
| PF13_0019 (Pf.13_1.110.0_CDS_at) | Sodium/hydrogen exchanger, Na+, H+ antiporter | 0.399 | 2.79E-04 |
| MAL13P1.163 (Pf.13_1.2.0_CDS_at) | ER lumen protein retaining receptor 1, putative | 0.632 | 6.38E-04 |
| PFL0885w (Pf.12.330.0_CDS_at) | Adaptor protein subunit, putative | 0.656 | 4.16E-04 |
| PFI0240c (Pf.9.302.0_CDS_at) | Cu2+ -transporting ATPase, Cu2+ transporter | 0.682 | 4.36E-04 |
| PFA0310c (Pf.1.20.0_CDS_at) | Calcium-transporting ATPase, putative | 0.697 | 8.71E-04 |
| **Heat shock, chaperone or stress response proteins** | | | |
| PFF1365c (Pf.6.40.0_CDS_at) | HECT-domain (ubiquitin-transferase), putative | 0.077 | 2.50E-04 |
| PFI0945w (Pf.9.228.0_CDS_at) | Thioredoxin, putative | 0.114 | 9.31E-04 |
| PFC0581w (Pf.3.147.0_CDS_at) | Co-chaperone p23, putative | 0.572 | 8.30E-04 |
| **Transcription, translation or nucleotide binding** | | | |
| PF13_0058 (Pf.13_1.84.0_CDS_a_at) | RNA binding protein, putative | 0.041 | 5.47E-06 |
| MAL8P1.70 (Pf.8.267.0_CDS_a_at) | Zinc finger C-x8-C-x5-C-x3-H type, putative | 0.041 | 1.13E-04 |
| PF10_0351 (Pf.10.530.0_at) | Probable protein, unknown function | 0.094 | 8.33E-04 |
| PF14_0119 (Pf.14.392.0_CDS_at) | p1/s1 nuclease, putative | 0.136 | 9.58E-04 |
| PF14_0173 (Pf.14.405.0_CDS_at) | Cyclic nucleotide-binding protein, putative | 0.157 | 3.23E-05 |
| PFF0200c (Pf.6.27.0_CDS_at) | Transcription factor with AP2 domain(s), putative | 0.161 | 4.28E-06 |
| PF13_0152 (Pf.13_1.261.0_CDS_at) | Transcriptional regulatory protein sir2 homologue | 0.196 | 3.98E-04 |
| PFE0895c (Pf.5.295.0_CDS_at) | Zinc finger protein, putative | 0.251 | 9.40E-04 |
| PF14_0196 (Pf.14.230.0_CDS_at) | Tetratricopeptide repeat family protein, putative | 0.301 | 2.50E-04 |
| MAL13P1.126 (Pf.13_1.307.1_a_at) | DHHC-type zinc finger protein, putative | 0.388 | 8.08E-04 |
| PF11_0091 (Pf.11.26.0_CDS_at) | Transcription factor with AP2 domain(s), putative | 0.493 | 6.49E-04 |
| chr7-tRNA-Thr-1 (Pf.7.290.0_CDS_at) | chr7-tRNA-Thr-1 | 0.494 | 2.74E-04 |
| PF08_0126 (Pf.8.59.0_CDS_at) | DNA repair protein rad54, putative | 0.641 | 4.78E-04 |
| PF10_0143 (Pf.10.31.0_CDS_at) | Transcriptional activator ADA2, putative | 0.726 | 7.34E-04 |
| **Exported, membrane or surface proteins** | | | |
| PFC0830w (Pf.3.163.0_CDS_at) | Trophozoite stage antigen | 0.030 | 1.34E-04 |
| MAL7P1.176 (Pf.7.64.0_CDS_s_at) | Erythrocyte binding antigen 175 | 0.039 | 3.23E-05 |
| PFD0110w (Pf.4.80.0_CDS_at) | Reticulocyte-binding protein homologue 1 | 0.053 | 2.88E-04 |
| PF10_0295 (Pf.10.83.0_CDS_at) | Conserved Plasmodium protein, unknown function | 0.059 | 9.08E-05 |
| MAL13P1.176 (Pf.13_1.610.0_CDS_at) | Reticulocyte binding protein 2, homolog b | 0.063 | 7.31E-04 |
| MAL13P1.60 (Pf.13_1.179.0_CDS_at) | Erythrocyte binding antigen-140 | 0.064 | 7.06E-05 |
| PF10_0346 (Pf.10.46.0_CDS_at) | Merozoite surface protein 6 | 0.067 | 2.77E-04 |
| PFF0870w (Pf.6.87.0_CDS_at) | Conserved Plasmodium membrane protein, unknown function | 0.067 | 4.07E-04 |
| PF13_0198 (Pf.13_1.161.0_CDS_at) | Reticulocyte binding protein 2 homolog A | 0.094 | 2.77E-04 |
| PFA0125c (AF461096.1_s_at) | Erythrocyte binding antigen-181 | 0.102 | 2.23E-04 |
| PFD0955w (Pf.4.4.0_CDS_at) | Apical merozoite protein | 0.103 | 3.98E-04 |
| PF10_0352 (Pf.10.128.0_CDS_at) | Merozoite surface protein | 0.107 | 4.28E-06 |
| PFB0120w (Pf.2.108.0_CDS_at) | Early transcribed membrane protein 2, ETRAMP2 | 0.108 | 8.77E-05 |
| PF14_0607 (Pf.14.243.0_CDS_at) | Conserved Plasmodium membrane protein, unknown function | 0.109 | 2.50E-04 |
| MAL7P1.6 (Pf.7.151.0_CDS_at) | Plasmodium exported protein (hyp12), unknown function | 0.110 | 9.08E-05 |
| PFD0100c (Pf.4.189.0_CDS_at) | Surface-associated interspersed gene 4.1, (SURFIN4.1) | 0.111 | 1.67E-04 |
| PF14_0325 (Pf.14.327.0_CDS_at) | Conserved Plasmodium membrane protein, unknown function | 0.115 | 1.40E-04 |
| PFC0120w (Pf.3.1.0_CDS_s_at) | Cytoadherence linked asexual protein 3.1 | 0.124 | 5.78E-04 |
| PFL0870w (Pf.12.328.0_CDS_at) | Thrombospondin-related apical membrane protein | 0.130 | 1.32E-04 |
| PFL2520w (Pf.12.397.0_CDS_at) | Reticulocyte-binding protein 3 homologue | 0.131 | 9.26E-05 |
| PFB0300c (Pf.2.3.0_CDS_at) | Merozoite surface protein 2 precursor | 0.134 | 5.55E-06 |
| PFC0090w (Pf.3.130.0_CDS_at) | Plasmodium exported protein, unknown function | 0.134 | 1.85E-04 |
| PF14_0018 (Pf.14.59.0_CDS_at) | Plasmodium exported protein (PHISTb), unknown function | 0.136 | 4.89E-04 |
| MAL13P1.130 (Pf.13_1.8.0_CDS_at) | Conserved Plasmodium membrane protein, unknown function | 0.139 | 2.89E-04 |
| PF14_0732 (Pf.14.25.0_CDS_at) | Plasmodium exported protein (PHISTb), unknown function | 0.140 | 6.60E-05 |
| PFL0060w (Pf.12.291.0_CDS_at) | Plasmodium exported protein, unknown function | 0.153 | 6.47E-04 |
| PF10_0343 (Pf.10.6.0_CDS_at) | S-antigen | 0.160 | 2.87E-04 |
| PFI0025c (Pf.9.281.0_CDS_at) | Rifin | 0.161 | 9.78E-04 |
| PF10_0345 (Pf.10.528.0_at) | Merozoite surface protein 3 | 0.162 | 1.65E-04 |
| PFD1150c (Pf.4.117.0_CDS_at) | Reticulocyte binding protein homolog 4, Rh4 | 0.162 | 6.63E-04 |
| PF10_0281 (Pf.10.72.0_CDS_at) | Merozoite TRAP-like protein, MTRAP | 0.163 | 2.36E-05 |
| PF10_0348 (Pf.10.30.0_CDS_at) | Erythrocyte membrane protein, putative | 0.165 | 5.61E-05 |
| PFI0845w (Pf.9.68.0_CDS_at) | Conserved Plasmodium membrane protein, unknown function | 0.174 | 7.70E-04 |
| PFD0295c (Pf.4.5.0_CDS_at) | Apical sushi protein, ASP | 0.176 | 3.13E-04 |
| PF10_0248 (Pf.10.146.0_CDS_at) | Conserved Plasmodium membrane protein, unknown function | 0.181 | 2.64E-04 |
| PF11_0040 (Pf.11.394.0_CDS_at) | Early transcribed membrane protein 11.2, etramp11.2 | 0.184 | 9.08E-05 |
| PFF0995c (Pf.6.295.0_CDS_at) | Merozoite surface protein 10, MSP10 | 0.187 | 2.77E-04 |
| PF11_0039 (Pf.11.170.0_CDS_at) | Early transcribed membrane protein 11.1, etramp11.1 | 0.191 | 3.23E-05 |
| PFB0105c (Pf.2.90.0_CDS_at) | Plasmodium exported protein (PHISTc), unknown function | 0.206 | 3.50E-04 |
| PFF1590w (Pf.6.245.0_CDS_at) | Rifin | 0.214 | 8.08E-04 |
| PFL1840w (Pf.12.35.0_CDS_at) | Conserved Plasmodium membrane protein, unknown function | 0.225 | 7.08E-04 |
| PFI1475w (Pf.9.6.0_CDS_s_at) | Merozoite surface protein 1 precursor | 0.235 | 3.52E-04 |
| PFI1730w (AF288172.1_s_at) | Cytoadherence linked asexual protein 9(CLAG9) | 0.235 | 1.12E-04 |
| PFA0010c (Pf.1.109.0_CDS_x_at) | Rifin | 0.243 | 9.69E-04 |
| PF14_0092 (Pf.14.37.1_CDS_a_at) | Conserved Plasmodium membrane protein, unknown function | 0.244 | 2.05E-04 |
| PFA0010c (Pf.1.109.0_CDS_at) | Rifin | 0.249 | 4.27E-04 |
| PFI1560c (Pf.9.59.0_CDS_at) | Conserved Plasmodium membrane protein, unknown function | 0.250 | 3.46E-04 |
| PFB0926c (Pf.2.40.0_CDS_s_at) | Plasmodium exported protein (hyp2), unknown function | 0.259 | 2.05E-04 |
| PFB0770c (Pf.2.27.0_CDS_at) | Conserved Plasmodium membrane protein, unknown function | 0.279 | 6.30E-04 |
| PF14_0753 (Pf.14.366.0_CDS_at) | Plasmodium exported protein (hyp13), unknown function | 0.282 | 3.66E-04 |
| PFD1110w (Pf.4.68.0_CDS_at) | Conserved Plasmodium membrane protein, unknown function | 0.286 | 4.78E-04 |
| PFL0010c (Pf.12.417.0_CDS_at) | Rifin | 0.299 | 7.72E-04 |
| PFB0310c (Pf.2.10.0_CDS_a_at) | Merozoite surface protein 4 | 0.309 | 3.57E-04 |
| PF10_0135 (Pf.10.157.0_CDS_at) | Conserved Plasmodium protein, unknown function | 0.324 | 9.15E-04 |
| MAL13P1.2 (Pf.13_1.546.0_CDS_at) | Rifin | 0.341 | 8.86E-04 |
| PFL1305c (Pf.12.183.0_CDS_at) | Conserved Plasmodium protein, unknown function | 0.419 | 3.71E-04 |
| PF10_0110 (Pf.10.239.1_at) | Conserved Plasmodium membrane protein, unknown function | 0.482 | 6.47E-04 |
| PF14_0215 (Pf.14.416.0_CDS_at) | Conserved Plasmodium membrane protein, unknown function | 0.499 | 3.09E-04 |
| PF14_0572 (Pf.14.717.0_CDS_at) | Conserved Plasmodium membrane protein, unknown function | 0.505 | 7.19E-04 |
| PF14_0653 (Pf.14.742.0_CDS_at) | Derlin-2, putative | 0.524 | 6.13E-04 |
| PFB0305c-a (Pf.2.47.0_CDS_at) | Merozoite surface protein 5 | 0.527 | 3.03E-04 |
| PFD0255w (Pf.4.8.0_CDS_a_at) | Ag-1 blood stage membrane protein homologue | 0.558 | 7.59E-04 |
| PF11_0467 (Pf.11.243.0_CDS_at) | Conserved Plasmodium protein, unknown function | 0.599 | 9.31E-04 |
| **Other metabolism** | | | |
| PFE0370c (Pf.5.150.0_CDS_at) | Subtilisin-like protease 1 | 0.021 | 4.07E-04 |
| PFI0540w (Pf.9.36.0_CDS_at) | Conserved Plasmodium protein, unknown function | 0.034 | 1.28E-04 |
| PF08_0108 (Pf.8.207.0_CDS_at) | Plasmepsin X | 0.056 | 3.23E-05 |
| PF10_0344 (Pf.10.174.0_CDS_at) | Glutamate-rich protein | 0.065 | 7.06E-05 |
| PF10_0094 (Pf.10.24.0_CDS_at) | Tubulin-tyrosine ligase, putative | 0.097 | 9.17E-05 |
| MAL8P1.150 (Pf.8.96.0_CDS_at) | Conserved Plasmodium protein, unknown function | 0.128 | 1.50E-04 |
| PFE1415w (Pf.5.142.0_CDS_at) | Cell cycle regulator with zn-finger domain, putative | 0.144 | 4.88E-06 |
| PFA0440w (Pf.1.84.0_CDS_at) | Photosensitized INA-labeled protein 1, PhIL1, putative | 0.149 | 7.90E-04 |
| PF14_0222 (Pf.14.200.0_CDS_at) | Ankyrin, putative | 0.155 | 4.02E-04 |
| PFL1565c (Pf.12.39.0_CDS_at) | CG2-related protein, putative | 0.177 | 3.41E-04 |
| PFE0340c (Pf.5.8.0_CDS_at) | Rhomboid protease ROM4 | 0.188 | 3.09E-04 |
| PF10_0220 (Pf.10.57.0_CDS_at) | Phospholipid scramblase 1, putative | 0.194 | 2.79E-04 |
| PFI1005w (Pf.9.114.0_CDS_at) | ADP-ribosylation factor-like protein | 0.204 | 7.29E-04 |
| PFE0395c (Pf.5.18.0_CDS_at) | 6-cysteine protein, putative | 0.215 | 2.21E-04 |
| PF11_0381 (Pf.11.195.0_CDS_at) | Subtilisin-like protease 2 | 0.230 | 1.97E-04 |
| MAL13P1.118 (Pf.13_1.141.0_CDS_at) | 3',5'-cyclic nucleotide phosphodiesterase | 0.272 | 5.64E-04 |
| PFB0345c (Pf.2.37.0_CDS_at) | Serine repeat antigen 4 (SERA-4) | 0.277 | 2.07E-04 |
| PF11_0395 (Pf.11.237.0_CDS_at) | Guanylyl cyclase | 0.279 | 1.37E-04 |
| PF14_0281 (Pf.14.50.0_CDS_at) | Plasmepsin IX | 0.286 | 1.30E-04 |
| PFC0160w (Pf.3.131.0_CDS_at) | Binding protein, putative | 0.301 | 5.78E-04 |
| PF10_0071 (Pf.10.187.0_CDS_at) | rhoGAP GTPase, putative | 0.303 | 2.99E-04 |
| PFB0350c (Pf.2.48.0_CDS_at) | Serine repeat antigen 3 (SERA-3) | 0.313 | 2.50E-04 |
| PF14_0420 (Pf.14.462.0_CDS_at) | Calmodulin-like protein | 0.315 | 3.18E-04 |
| PFL1870c (Pf.12.271.0_CDS_at) | Sphingomyelin phosphodiesterase, putative | 0.324 | 9.31E-04 |
| MAL8P1.81 (Pf.8.54.0_CDS_at) | Phosphopantothenoylcysteine decarboxylase, putative | 0.335 | 4.28E-06 |
| PF10_0268 (Pf.10.1.0_CDS_x_at) | Merozoite capping protein 1 | 0.338 | 8.56E-04 |
| PFL1780w (Pf.12.364.0_CDS_at) | Protein-S-isoprenylcysteine O-methyltransferase, putative | 0.390 | 6.23E-04 |
| PFD0660w (Pf.4.45.0_CDS_a_at) | Phosphoglycerate mutase, putative | 0.401 | 3.41E-04 |
| PFD0390c (Pf.4.74.0_CDS_at) | AAA family ATPase, putative | 0.420 | 3.57E-04 |
| PFI0970c (Pf.9.8.0_CDS_at) | TLD domain-containing protein | 0.440 | 8.84E-04 |
| PFF0615c (Pf.6.101.1_a_at) | 6-cysteine protein, putative | 0.450 | 1.69E-04 |
| PF10_0020 (Pf.10.55.0_CDS_at) | Alpha/beta hydrolase, putative | 0.494 | 2.05E-04 |
| PF14_0246 (Pf.14.135.0_CDS_at) | Phosphoenolpyruvate carboxylase, putative | 0.507 | 7.22E-04 |
| PF14_0159 (Pf.14.620.0_CDS_a_at) | Root hair defective 3 GTP-binding protein (RHD3) homolog, putative | 0.513 | 6.46E-04 |
| PFI1255w (Pf.9.148.0_CDS_at) | Zinc-binding protein (Yippee), putative | 0.519 | 5.30E-04 |
| PFL1330c (Pf.12.47.0_CDS_at) | Cyclin-related protein, Pfcyc-2 | 0.530 | 2.52E-04 |
| PF14_0363 (Pf.14.151.0_CDS_x_at) | Metacaspase-like protein | 0.560 | 9.40E-04 |
| PF10_0311 (Pf.10.276.0_CDS_at) | Protein phosphatase inhibitor, putative | 0.763 | 9.66E-04 |
